# Evolutionarily Stable Attenuation by Genome Rearrangement in a Virus

**DOI:** 10.1534/g3.113.006403

**Published:** 2013-08-01

**Authors:** Nicole Cecchini, Matthew Schmerer, Ian J. Molineux, Rachael Springman, James J. Bull

**Affiliations:** * Department of Integrative Biology, The University of Texas at Austin, Austin, Texas 78712; † Center for Computational Biology and Bioinformatics, The University of Texas at Austin, Austin, Texas 78712; ‡ Department of Molecular Biosciences, The University of Texas at Austin, Austin, Texas 78712; § Institute for Cellular and Molecular Biology, The University of Texas at Austin, Austin, Texas 78712

**Keywords:** genome, engineering, attenuation, adaptation, virus, synthetic biology

## Abstract

Live, attenuated viruses provide many of the most effective vaccines. For the better part of a century, the standard method of attenuation has been viral growth in novel environments, whereby the virus adapts to the new environment but incurs a reduced ability to grow in the original host. The downsides of this approach were that it produced haphazard results, and even when it achieved sufficient attenuation for vaccine production, the attenuated virus was prone to evolve back to high virulence. Using bacteriophage T7, we apply a synthetic biology approach for creating attenuated genomes and specifically study their evolutionary stability. Three different genome rearrangements are used, and although some initial fitness recovery occurs, all exhibit greatly impaired abilities to recover wild-type fitness over a hundred or more generations. Different degrees of stable attenuation appear to be attainable by different rearrangements. Efforts to predict fitness recovery using the extensive background of T7 genetics and biochemistry were only sometimes successful. The use of genome rearrangement thus offers a practical mechanism of evolutionary stable viral attenuation, with some progress toward prediction.

Engineered modifications of a genome are readily understood to have fitness costs (Sleight *et al.* 2010; Yang *et al.* 2013). Yet these fitness costs may be transitory because compensatory evolution during long-term growth frequently restores much of the lost fitness (Bull *et al.* 2007; Hanley 2011). An ability to predict or contain long-term fitness recovery in response to an engineered modification would benefit many applications in which the intent is to release the engineered organism as a free-living population. For example, fitness recovery is undesirable with attenuated vaccines and with transgenic animals/plants that might escape into natural populations. In a specific case that motivates our study, global poliovirus eradication remains elusive because the live, attenuated Sabin vaccine strains easily evolve to recover high virulence (Nathanson and Kew 2010; Kew 2012). Knowing how to engineer stable attenuation (permanent, low fitness) might lead to a vaccine more conducive to eradication.

Bacteriophage T7 has become a model system for systems biology, synthetic biology, and evolution of viruses. After decades of genetic and biochemical work on T7 (Dunn and Studier 1983; Molineux 2006) an empirically parameterized virtual model of its infection cycle was developed (Endy *et al.* 2000; Kosuri *et al.* 2007), and various types of engineered genomes have been tested (Endy *et al.* 2000; Bull and Molineux 2008); a cell-free system for growing T7 *in vitro* promises even further advances (Shin *et al.* 2012). The combined approaches have been integrated with the hope of predicting not only the consequences of engineering but also of predicting evolution in response to engineering. The virus is highly evolvable, and many engineered changes that initially cause major fitness defects are largely overcome when the virus is grown for as little as a few hundred generations (Bull and Molineux 2008). However, preliminary evidence suggests that some types of engineered changes—genome rearrangements —are evolutionarily stable and result in long-term fitness reductions. This example raises the possibility that simple engineering changes may be able to permanently cripple genomic fitness, thus allowing us to produce attenuated organisms that cannot recover original fitness in any moderate period of outgrowth.

The present study explores in-depth the evolution of rearranged T7 genomes to test their stability against evolution toward high fitness. Genome organization is a major determinant of T7 fitness (here, measured as growth rate) because the linear entry of the genome ties gene position to the timing of gene expression. The questions motivating our work are whether and which genome rearrangements present insurmountable obstacles to the recovery of high fitness through compensatory evolution and whether we can predict evolutionary pathways.

## Methods

### Strains and media

The basic phage strains are described in Table 1A and illustrated in Table 1B. All are from the collection of I.J.M. or J.J.B. All phage growth was conducted at 37° and used host IJ1133 (*Escherichia coli* K-12 Δ*lacX74 thi* Δ(*mcrC-mr*r)*102*::Tn*10*). LB broth was 10 g of NaCl, 10 g of Bacto tryptone, and 5 g of Bacto yeast extract per liter. Plates used LB broth with 1.5% Bacto agar. Determinations of phage titers used plates overlaid with soft agar (0.7% Bacto agar in LB) containing a suitable density of hosts.

**Table 1:** Phage strains used

A. Notation and descriptions			
Notation	Genotype; Origin	Purpose	Reference
T7:WT	Wild-type (Genbank V01146)	Control for rearranged genomes	(Dunn and Studier 1983)
T7:12(1)13	T7 with gene *1* translocated between genes *12* and *13*; genes *0.5−0.7* deleted. Originally developed to test virtual model of T7 based on a predicted long life cycle delay.	Adapted to IJ1133 for 150 generations in prior work; used here for recombination against T7:12(Plac-1)13 and brief outgrowth	Δ*0.5-1* in (Springman *et al.* 2005); Ecto*12* in (Endy *et al.* 2000).
T7:12(Plac-1)13	T7:12(1)13 with 1.5 kb of *E. coli* DNA inserted in front of the ectopic gene *1*; the insert contains *lac* I and the lac promoter; T7 is deleted for early region elements A1-gene *0.7*. Originally developed to study genome entry and expression in the absence of the early promoters.	Evolved to evaluate the benefit of an *E. coli* promoter immediately upstream of ectopic gene *1*	D394-5904:plac1 in (García and Molineux 1996); 4101plac*1* in (Struthers-Schlinke *et al.* 2000)
T7:3.8(1)3.8	T7 with gene *1* translocated into gene *3.8*. Originally developed to test virtual model of T7 based on a predicted short life cycle delay.	Evolved to evaluate effect of a new ectopic location for gene *1* on long term fitness	Ecto*3.8* in (Endy *et al.* 2000)
T7:LME	T7 with a reciprocal exchange of *φ13−φ17* and *φ1.1B−φ2.5*; gene *1* is in the wild-type location. LME refers to a gene order of “late,” “middle,” and “early,” although not all early and late genes are misplaced. Obtained as a spontaneous beneficial mutant during adaptation of T7:12(1)13.	Evolved to evaluate the long-term fitness stability of a rearranged genome in which gene *1* is in its wild-type location	Δ*1*Be in (Springman *et al.* 2005)

B. Schematic of phage strain genomes			
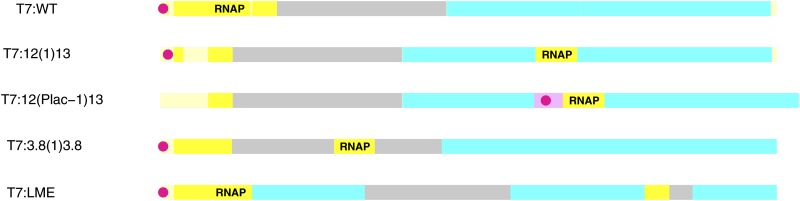

Each colored bar represents the T7 genome; yellow indicates early genes, gray represents middle genes, and light blue represents late genes; gaps in the early region are deletions. RNAP indicates the approximate location of the RNA polymerase gene (gene *1*). The red dot represents *E. coli* promoter sequences, and the pink segment in T7:12(Plac-1)13 in front of RNAP is 1.5kb of sequence from the *E. coli* lac operon. The relative scale is approximate. Subsequent figures provide more detail about genome content and gene location.

### Growth of phage for adaptation

The adaptations selected rapid phage growth. Adaptation to IJ1133 in LB broth used serial transfer, as described elsewhere (Heineman and Bull 2007; Bull *et al.* 2011). To summarize in brief, cells from a frozen aliquot were added to 10 mL of broth in a 125-mL flask, grown for 1 hr to a density of approximately 10^8^ cells/mL, after which 10^4^−10^6^ phage were added. Cultures were grown with phage for a predetermined time, whence an aliquot was added directly to a new flask containing cells also grown 1 hr. Cultures were sometimes allowed to proceed to lysis, ensuring recombination from the multiple infections. The duration of an adaptation in hours was converted to an approximate number of generations based on the lysis time of the phage; duration (in generations, G) is indicated by a _G following the strain designation.

Adaptation of T7:LME was carried out in three stages. The first 200 generations were carried out by serial transfer and reported in Springman *et al.* (2005). The initial phage carried a partial deletion of gene *1* (RNAP) at the wild-type location but carried an ectopic copy displaced 23 kb; within a few generations, recombination restored gene *1* to its wild-type location but reciprocally exchanged several early genes with late genes. Adaptation for the remainder of the 200 generations retained that new gene order. Here, that adapted phage (T7:LME_200) was subjected to an extensive additional adaptation that consisted of two phases. The initial phase was growth in a two-stage chemostat in which actively growing IJ1133 from a source tube was fed continuously into a tube containing phage (*e.g.*, Wichman *et al.* 2005). After several hundred hours of chemostat growth, the second phase began with a recombination between the final chemostat population (T7:LME_1940) and the starting phage (T7:LME_200); the recombinant pool was grown by serial transfer for another 60 generations to yield T7:LME_2000. The serial transfer phase of the recombinant mix purges mutations that evolved under continuous culture but are detrimental in the serial transfer regime (Nguyen *et al.* 2012).

### Fitness assays

Fitness was measured in the serial transfer environment, the main difference being that phage densities in fitness assays are maintained at least 10-fold lower than cell densities throughout to ensure that hosts are never limiting. Fitness is given as doublings per hour, an absolute growth rate that applies regardless of phage generation time.

### Fitness calculations from life history parameters

In one study, we estimated the fitness effect of a promoter that accelerated phage lysis on a genomic background whose fitness and lysis time approximated that reported for the evolved phage Δ0.5-1e in Springman *et al.* (2005). The fitness equation r = kC(b e^−Lr^ −1) was taken from Bull (2006) and required inputs of cell density (C = 1 × 10^8^/mL), adsorption rate (k = 5 × 10^−9^ mL/min), lysis time (L = 19 min), and burst size (b = 300) to calculate the rate of growth, *r*, which when multiplied by 60/ln(2) gives doublings/hr, the measure of fitness used here. The fitness calculated for these parameters was 24.2, matching the value reported by Springman *et al.* (2005). The fitness benefit of a reduced lysis time was estimated by recalculating fitness with all parameters held constant except lysis time. To achieve a fitness value of 24, the lysis time was reduced 2 min from that reported by Springman *et al.* (2005) (21 min); the fitness effects of the mutant genotypes were not highly sensitive to such small variations in parameter values.

### Eclipse time assays

Eclipse time (the time from infection of a cell to the first intracellular appearance of infectious progeny) was measured by infecting cells in the fitness assay environment and extracting undiluted samples over chloroform at one-minute intervals; the cells were subjected to freeze-thaw before plating to enhance disruption of cell integrity. The eclipse period was indicated by the earliest statistically significant increase in titer. Two-three independent assays were conducted for each phage.

### Sequences

DNA was obtained from phage by phenol extraction and subjected to ‘454’ pyrosequencing (Illumina in one case). Reads were mapped against a template with the program *Breseq* (http://barricklab.org/breseq; Barrick *et al.* 2009). Template genome files are uploaded in fasta format in Supporting Information, File S1; fastq files of the sequences are uploaded as Genbank files (SRS429977 for T7:LME_200; SRS429978 for T7:LME_2000; SRS430093 for T7:12(Plac-1)13_80; SRS430095 for T7:3.8(1)3.8).

## Results

The three studies conducted here address different aspects of genome reorganization as a method to achieve long-term attenuation with the virus T7. Our emphasis is on both the evolutionary stability of attenuation, *i.e.*, whether adaptive evolution reverses the attenuation, and on our ability to predict the evolutionary response to the reorganization. For prediction, we have a wealth of biochemical understanding of this phage (Dunn and Studier 1983; Molineux 2006) as well as a virtual model of the infection cycle (Endy *et al.* 2000; Kosuri *et al.* 2007). Each study uses a different modified T7 genome, selects it for fast growth, and monitors fitness and sequence evolution.

### No benefit of a predicted recovery pathway in a reorganized genome

#### Background and predictions:

A central element in the speed of the T7 life cycle is the time from infection to expression of the RNA polymerase (RNAP) gene. The expression of all other essential genes requires the phage RNAP, so any delays in its expression should delay all downstream events, including lysis and generation time. Genomes with displacements of the RNAP gene were used as the empirical basis for testing a virtual model of the T7 infection cycle (Endy *et al.* 2000). For large displacements, a substantial depression in fitness (phage growth rate) is predicted because of long delays in entry of the RNAP. For short displacements, the predictions are complicated by auto-expression feedback loops of the RNAP (Endy *et al.* 2000, and see the section *RNAP gene displacement affects the fitness limit quantitatively but defies prediction*).

**Figure 1:**
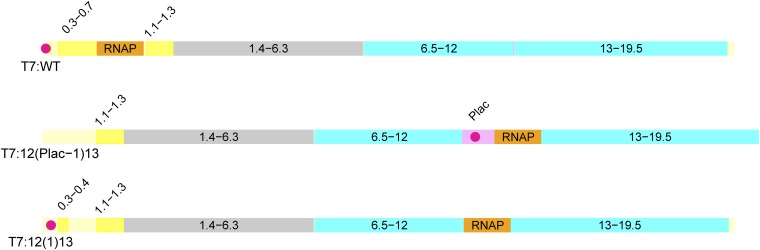
Gene order and key regulatory element locations for wild-type T7 and two genomes with phage RNAP genes displaced to the region between late genes *12* and *13*; genome identity is labeled under the front end of each color-coded bar. The color coding is yellow for early genes, gray for middle (DNA metabolism), and light blue for late (morphogenetic functions), the RNAP gene (which is an early gene) is shown in orange to highlight its position; deletions of early-region genes are shown in ivory, but as they are considered to have minor effects in comparison to the engineering, they are indistinct. The red dot represents *E. coli* promoter sequences (three clustered native promoters in phages T7:WT and T7:12(1)13, 1 engineered promoter in T7:12(Plac-1)13). In T7:12(Plac-*1*)13, the ectopic RNAP gene is preceded by 1.5 kb of *E. coli* DNA containing the *lac* I gene as well as the lac promoter/operator (pink). All the native left-end *E. coli* promoter sequences on the T7:12(Plac-1)13 genome are deleted; only ~400 bp of noncoding DNA, including the 160-bp terminal repeat, precede gene *1.1*. In contrast, the phage RNAP gene of T7:12(1)13 is displaced with no adjacent *E. coli* sequence, and the native left-end phage sequences that encode *E. coli* promoter sequences are intact.

Our focus in this section is T7:12(1)13, a genome whose RNAP gene is displaced by approximately 50% of the genome length (genome B in Figure 1, Endy *et al.* 2000). On a log scale, fitness (doublings/hr) of the initial virus is approximately two thirds that of wild-type (Springman *et al.* 2005), broadly consistent with computational predictions (Endy *et al.* 2000). Given that we can at least qualitatively understand the fitness consequences of this rearrangement, what are the expected evolutionary pathways of its fitness recovery?

Predicting the evolutionary response to this rearrangement requires some understanding of genome entry mechanisms. The timing of RNAP expression in T7 can be described as occurring in two steps, genome entry and transcription. Most of a wild-type genome is internalized by transcription, but the initial ~1000 bp enter at 140 nt/sec by a process that requires the ejected virion proteins gp15 and gp16 (García and Molineux 1996; Struthers-Schlinke *et al.* 2000; Kemp *et al.* 2004; Chang *et al.* 2010). That portion of the genome contains three host-specific promoters, A1, A2, and A3, and transcription from those promoters then brings in the next portion of the genome, including the entire RNAP gene, designated gene *1* because it is the first essential gene to enter the cell. Host transcription proceeds to the terminator TE, just beyond 7.5 kb, and the remainder of the genome is then brought in by transcription using T7 RNAP.

In T7:12(1)13, the ectopic RNAP gene (gene *1*) is displaced approximately 23 kb from its wild-type location. Transcription by *E. coli* RNAP initiated at A3 occurs at 70 bases/s at 37° (Kemp *et al.* 2004) and would reach the ectopic gene *1* in just over 5 min if not blocked by TE. The efficiency of termination by TE, however, is substantial (Molineux 2006). Thus, the most immediate impediment to expression of RNAP is TE, and abolishing its termination is predicted to be the first major step in fitness recovery. Adaptations of these genomes did indeed exhibit single-base changes in TE that led to profound improvements in fitness (Springman *et al.* 2005). One of these evolved genomes, T7:12(1)13_150, is referenced below in: *Evolution of a recombinant mix that carries promoters in two locations*.

With TE termination abolished, expression of the ectopic gene *1* becomes physically coupled with its entry because the host RNAP now serves both functions simultaneously; gene *1* is expressed by the host RNAP as the RNAP pulls the gene into the cell. Genomes lacking termination by TE still experience a pronounced delay in phage RNAP expression, however, because 330 sec is needed for host RNAP to reach the ectopic gene *1*. An alternative pathway, one involving two evolutionary steps, can theoretically accelerate expression of gene *1* and thus provide a fitness advantage. In this two-step pathway, genome entry becomes decoupled from phage gene expression.

Evolutionarily, T7 genome entry is not tied to host RNAP. Mutations affecting phage protein gp16 cause full genome entry and do so at 140 bp/sec (Kemp *et al.* 2004), twice the rate of entry by host RNAP. Thus a phage with this entry mutation would accelerate entry of the ectopic gene *1* to just 160 sec after entry of A1-A3, nearly 3 min faster than under host RNAP control. If transcription of the ectopic gene *1* could begin immediately after it entered the cell—as from a promoter placed immediately upstream—the life cycle could begin nearly 3 min earlier.

The advantage of a 3-min reduction in the life cycle is substantial, approximately 4 dbl/hr (see *Methods*). The predicted benefit is reduced somewhat if a cryptic *E. coli* promoter near gene *10* is active: with the rapid entry mutation, expression of the ectopic gene *1* from that promoter could begin at 235 sec (128 sec to entry of this cryptic promoter and 107 sec for transcription to reach gene *1*). The life cycle acceleration from a promoter at gene *1* is now 75 sec, with a predicted fitness benefit of 1.6 dbl/hr. (This cryptic promoter is known to function on plasmids but activity is not known on the phage genome, Rosenberg *et al.* 1987, hence our uncertainty) Regardless of whether the cryptic promoter is active, the phage requires a second “mutation” that enacts promoter activity immediately in front of the ectopic gene *1* to realize the full benefit of faster entry. Yet, although phages with ectopic gene *1* evolved rapid entry, no changes could be interpreted as effecting a new promoter (Springman *et al.* 2005). It is entirely plausible, however, that mutational constraints prevented this second evolutionary step because multiple mutations could be required simultaneously to cause any increase in transcription.

We thus used engineering to determine the possible benefit of an *E. coli* promoter adjacent to gene *1*. The phage T7:12(Plac-*1*)13 carries *lac* I followed by the *lac* promoter as a 1.5kb insert upstream of the ectopic gene *1*. This phage experiences delayed entry from the lack of all left end *E. coli* promoters (see Table 1A; at 30º, its eclipse period is 30 min longer than that of T7:WT; Struthers-Schlinke *et al.* 2000). However, mutations in gene *16* are readily selected to allow complete transcription-independent genome entry at 140 bp/s at 37° (García and Molineux 1996; Struthers-Schlinke *et al.* 2000). If our understanding of the critical determinants of gene expression in this phage is correct, the fitness of T7:12(Plac-*1*)13, once it has acquired a rapid-entry mutation, should exceed that of evolved T7:12(1)13 genomes.

#### Evolution of T7:12(Plac-1)13:

Adaptation of T7:12(Plac-*1*)13 for ~80 generations (27 hr) resulted in several expected changes. (1) Rapid entry evolved, based on observed eclipse time comparisons between a known entry mutant and the initial phage (data not shown). Curiously, the only substitutions observed in entry proteins (gp15 L15F and gp16 E1236G) are not in domains known to cause early entry in other studies (Struthers-Schlinke *et al.* 2000). (2) No mutation in the terminator TE was observed; none is expected because there are no left-end *E. coli* promoters in this phage. (3) The *lac* I gene was lost but the *lac* promoter remained. *lac* I offers no benefit and may even delay gene *1* expression.

**Figure 2:**
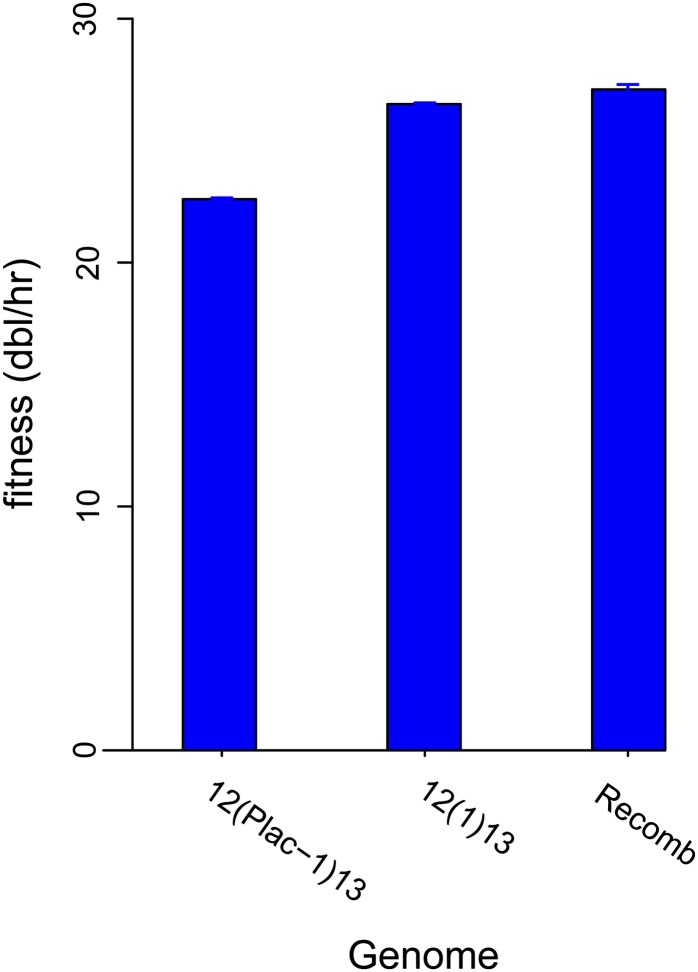
Evolved fitnesses of genomes with ectopic placement of gene *1* (RNAP) between genes *12* and *13* but differing in locations of *E. coli* promoter sequences. For visual clarity, each genome label on the horizontal axis omits the index indicating the number of generations of evolution (they are respectively _80, _150, and _30). In T7:12(Plac-1)13, the left end promoters A1-A3 are absent, and the lac promoter is placed adjacent to the ectopic gene *1*. In T7:12(1)13, the left end promoters are present but no ectopic promoter is present. The Recomb is a pooled recombinant mix of those two genomes evolved 30 generations, long enough to let the best combination of *E. coli* promoters ascend. Error bars of 1 SE are shown.

Given these successes in predicting evolution at the genomic level, the singular surprising outcome was a low final fitness. Fitness of the evolved T7:12(Plac-*1*)13_80 was 22.6 dbl/hr, fully 3 dbl/hr below that of the previously evolved T7:12(1)13_150 (Figure 2, Springman *et al.* 2005, and see the next paragraph).

#### Evolution of a recombinant mix that carries promoters in two locations:

The unexpected low fitness of T7:12(Plac-*1*)13_80 led us to consider that the genome design had possibly constrained its evolution. As T7:12(Plac-1)13 differs from T7:12(1)13 in more than just the ectopic *E. coli* promoter, an additional test was done to enable the evolution of a new promoter directly in T7:12(1)13. T7:12(Plac-*1*)13_80 and the previously evolved T7:12(1)13_150 were recombined against each other. The recombinant pool was grown by serial transfer for nearly 30 generations. The recombinant pool would have started with all combinations of genomes, some with left end promoters A1-A3 present, others with those absent, and similarly for Plac. If the lack of native left end promoters in T7:12(Plac-*1*)13 was an impediment to its fitness improvement, this mix would have allowed evolution of a phage with *E. coli* promoters in both locations. The outgrowth population retained the composition of T7:12(1)13, lacking the downstream *E. coli* promoter, as well as its approximate fitness (Figure 2; parallel fitness estimates were obtained as 27.1 ± 0.2 dbl/hr for the recombinant population and 26.5 ± 0.05 for T7:12(1)13_150).

#### Summary:

Neither adaptation supports the predicted benefit of a downstream *E. coli* promoter. Furthermore, the results offer only mixed support for our ability to predict evolution of these rearranged genomes. We can predict when a terminator mutation does and does not have an advantage and can sometimes predict when entry mutations and ectopic *E. coli* promoters have an advantage. However, we cannot predict even the qualitative fitness benefit of a two-step recovery pathway. The failure to evolve novel promoter activity in the original genome with displaced RNAP genome was apparently not due to mutation limitation.

### RNAP gene displacement affects the fitness limit quantitatively but defies prediction

#### Background and predictions:

The T7 virtual model of the intracellular life cycle is a kinetic model that incorporates parameters for more than 100 genetic elements (Endy *et al.* 2000; Kosuri *et al.* 2007). It is versatile, allowing the programmer to specify an arbitrary linear order of those elements during genome entry, with consequent effects on the timing of the function of those elements.

A broad-scale prediction of the model is that fitness declines as gene *1* is moved increasingly downstream from the wild-type location (Endy *et al.* 2000). This prediction holds over large displacements, but predicted fitnesses of genomes with gene *1* locations near the wild-type position vary inconsistently with distance because of auto-amplification feedback when the RNAP gene is placed immediately downstream of a T7 promoter. As an empirical test of the model, T7 genomes were created with the RNAP gene translocated to three different locations 3′ to the wild-type position (Endy *et al.* 2000). The most extreme displacement (23-kb, phage T7:12(1)13) exhibited a life cycle delay of ~40 min (at 30°), semiquantitatively consistent with the predicted delay of 30 min relative to wild-type. The two phage with lesser displacements were predicted to have almost imperceptible life cycle delays, but were observed in practice to have substantial delays relative to wild-type, as much as 10 min for T7:3.8(1)3.8 (at 30°). The computationally predicted near absence of a life cycle delay for this phage was interpreted by us as a prediction that adaptive evolution would fully overcome the initial fitness cost.

#### Evolution:

**Figure 3:**
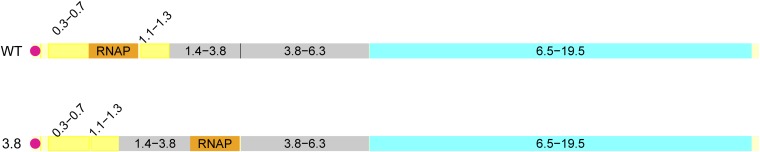
Displacement of the T7 RNAP gene in T7:3.8(1)3.8. Color coding is as in Figure 1.

**Figure 4:**
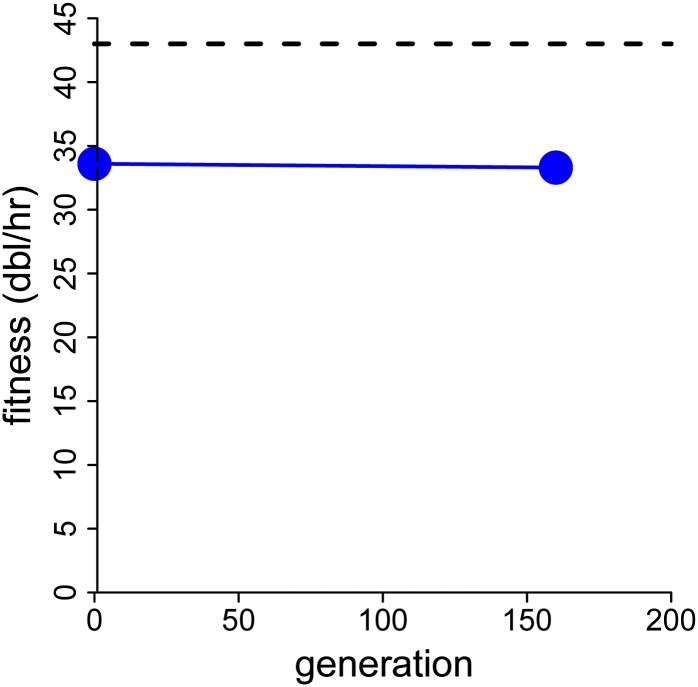
Adaptation of T7:3.8(1)3.8 for 160 generations occurred without a detectable fitness increase (blue dots connected with solid line). SEs of both points are 0.1, obscured by the points. Fitness of evolved wild-type is shown as a horizontal dashed line at 43.

We adapted T7:3.8(1)3.8, whose gene *1* displacement is 5.7 kb (Figure 3). Its fitness evolution did not match the predicted return to wild-type levels (Figure 4). From an initial fitness of 33.6 ± 0.1, the final fitness after ~240 generations of adaptation was 33.3 ± 0.1, not significantly different. (Sixty hours of adaptation were converted at four generations/hr based on an observed lysis time of ~15 min.) This fitness is intermediate between the fitness of 27 for the evolved T7:12(1)13_150 (reported above) and the 42.6 of evolved wild type (Bull *et al.* 2011).

Sequences of the population at ~160 gens (40 hr) identified two changes greater than 90% frequency and two others near 30% (Table 2). Two of these changes closely match substitutions observed in the published adaptation of T7:12(1)13 (Springman *et al.* 2005)—the TE change and the gene *16* change—although the nucleotide positions differ slightly for both. A reduction in termination efficiency at TE has obvious benefit because the ectopic location of the RNAP gene in T7:3.8(1)3.8 is downstream of TE. The benefit of the gene *16* change may be that there are cryptic *E. coli* promoters between A1−A3 and the site of the ectopic gene *1* in gene *3.8* (Dunn and Studier 1983). However, the fact that the frequency of the fast entry mutation in gene *16* is much less than 1.0 after 240 generations of adaptation suggests that its contribution to fitness is minor.

**Table 2:** Changes in T7:3.8(1)3.8_160*^a^*

Gene/Element	Function	Amino acid change	T7:WT Position (change)	Frequency
TE	Terminator		7576 (T->C)	1.0
*1.7*, *1.8*	Nucleotide kinase, unknown	A 196 T, M 1 I	8751 (G->A)	0.32
*16*	Fast entry	K 825 T	33068 (A->C)	0.29
*17*	Tail fiber, fast adsorption	T 118 A	34975 (A->G)	0.92

a Because so little evolution was observed, all substitutions greater than 25% are included here. The template used was based on the published description (Endy *et al.* 2000).

Inspection of sequences from the initial population of T7:3.8(1)3.8 revealed that the TE change was already present (observed in six of six isolates of the initial population; in contrast the gene *17* change was absent in six of six isolates). This observation both argues for the strong benefit, hence very rapid ascent, of the TE change and helps explain why no fitness gain was observed during the adaptation. When mutations have a strong benefit, there is in fact little that can be done to avoid their ascent during fitness assays, even when the starting isolate lacks them. It is thus unlikely that the true initial fitness of T7:3.8(1)3.8 can be determined accurately.

#### Summary:

Together, these first two experiments indicate that the genome is highly, apparently permanently, sensitive to the placement of the RNAP gene. These results point to a possible mechanism of permanent, quantitative attenuation by displacing this single gene to different locations. This is a practical observation, as we lack the ability to predict some aspects of fitness evolution in both systems.

### Attenuation persists 2000 generations in a rearrangement with wild-type RNAP location

#### Background and predictions:

**Figure 5:**
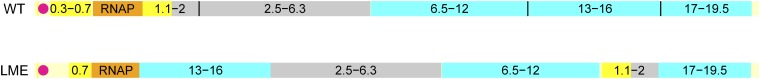
Genome organization in wild-type T7 (upper) compared with T7:LME (lower); colors as in Figure 1 and Figure 3. The reorganization in LME thus places late genes in the early-middle region and places early and middle genes in the late region, but the RNAP gene is in the wild-type location.

One of the prior adaptations of T7:12(1)13 evolved an unusual genome order as a response to the fitness cost of the ectopic gene *1* (Springman *et al.* 2005). This reorganization restored gene *1* to its wild-type location but exchanged nine early/middle genes with four late genes (phage T7:LME in Figure 5). Coincident with the reorganization, there was an immediate fitness increase of 11 dbl/hr, yet over the next ~180 generations of adaptation, fitness increased only 2.5 dbl/hr further, and final fitness remained depressed well below that of the evolved wild-type.

This unusual gene order allows us to investigate the evolutionary stability of attenuation when the elements known to influence genome entry are in the same locations as in wild-type. Although fitness remained depressed after 200 previous generations of adaptation and might be construed to imply stable attenuation, fitness had nonetheless increased modestly after the rearrangement. Furthermore, that modest fitness increase was accompanied by a surprising 16 point mutations. Molecular evolution was thus active, so it seems feasible that the phage might accelerate its fitness evolution if adapted for longer. The fact that gene *1* is in its wild-type location also reinforces our expectation that the attenuation may decay on long term evolution: all major fitness effects should stem from imbalances in expression of genes transcribed by the phage RNAP, and it is plausible that appropriate expression levels may re-evolve easily.

#### Evolution:

**Figure 6:**
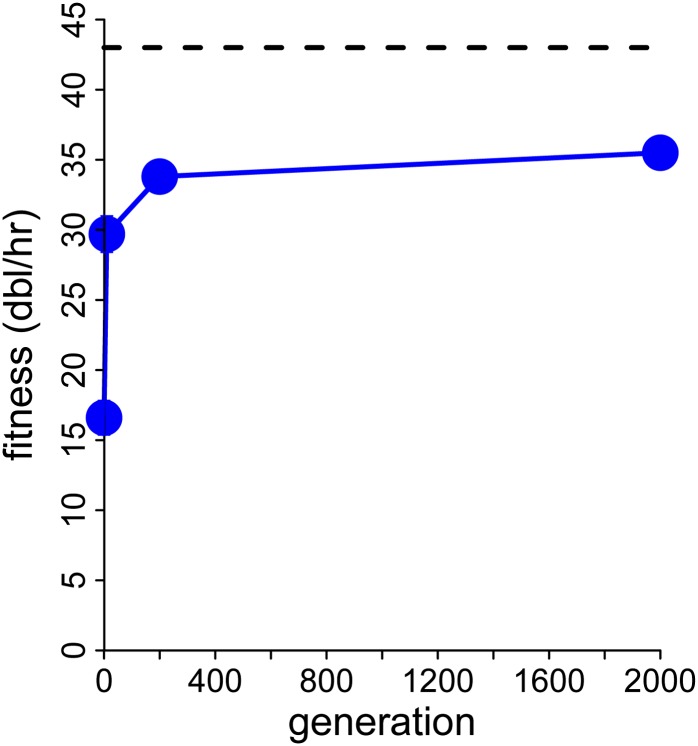
Fitness evolution of T7:LME (solid line). The dots indicate the measured fitness at various points during the continued adaptation of the virus; the lines merely connect the points. The first two steps of the adaptation were performed in prior work, and the fitnesses of the first two points are from that study (Springman *et al.* 2005). The final adaptation, from generation 200–2000, was performed here. Fitnesses of the last two points were estimated here (fitness of T7:LME_200 was re-estimated for the present study and is 1.7 above the value obtained by the methods in the 2005 study). Error bars are ± 1 SE, sometimes obscured by the point. Fitness of evolved wild-type is shown as a horizontal dashed line at 43.

Here, T7:LME_200 was evolved 1800 generations to yield T7:LME_2000. Final fitness was 35.5 ± 0.3 dbl/hr, a moderate increase from 33.8 ± 0.5 for the initial T7:LME_200 (Figure 6). Thus, the adaptation here was carried out approximately 10 times as long as the original adaptation (after the rearrangement) and yielded approximately the same fitness increase. Current fitness is approximately 7 dbl/hr below that attained by wild-type phage.

The consensus sequence of the population of T7_LME__2000 contained 17 mutations in addition to those found in T7:LME_200, indicating that evolution continued but that fitness effects per mutation were small by usual standards of T7 adaptation (Table 3). One change was a 154-bp tandem duplication in the intergenic region between the RNAP gene (*1*) and the transposed gene *13*; this duplication included the promoter φ13 and thus likely had important regulatory effects. Only one other change was in the transposed regions, and it was a non-coding change in gene *1.7*. Some other changes are in genes commonly observed to acquire substitutions during adaptation (the tail fiber gene *17* and the minor capsid gene *10B*), or affect functions that may plausibly be argued to alter the coordination of life cycle components (DNA and RNA metabolism).

**Table 3:** Significant genomic changes evolved in T7:LME_2000*^a^*

Gene/Element	Function	AA Change	Position*^b^*	T7:WT Position
*1*	RNA polymerase	Q 184 R	2513 (A→G)	3721
IG (genes *1*,*13*)	Regulatory	154 bp duplication	4630	No equivalent
*4.5*	DNA metabolism	N 18 D		
*5*	DNA polymerase	D 376 G	18089 (A→G)	15479
*8*	Baseplate	M 523 G	24416 (A→G)	21806
*10B*	Minor capsid	E 38 K	26698 (G→A)	24088
*12*	Tail	S 462 G	28835 (A→G)	26225
*17*	Tail fiber	K 211 T	33771 (A→C)	35255
*17*	“”	R 542 H	34764 (G→A)	36248
*18*	Terminase subunit	K 5 E	35081 (A→G)	36565

a Changes listed here were observed in the consensus sequence, thus at a frequency of at least 0.5. Changes not listed included four silent changes within genes (*4.3*, 5, *10B*, and *1.7*), two base changes in the 5′ noncoding region at bases 161 and 235, and a 13 base duplication at base 183. IG refers to an intergenic location.

b Position refers to the template for T7:LME_200.

#### Summary:

**Figure 7:**
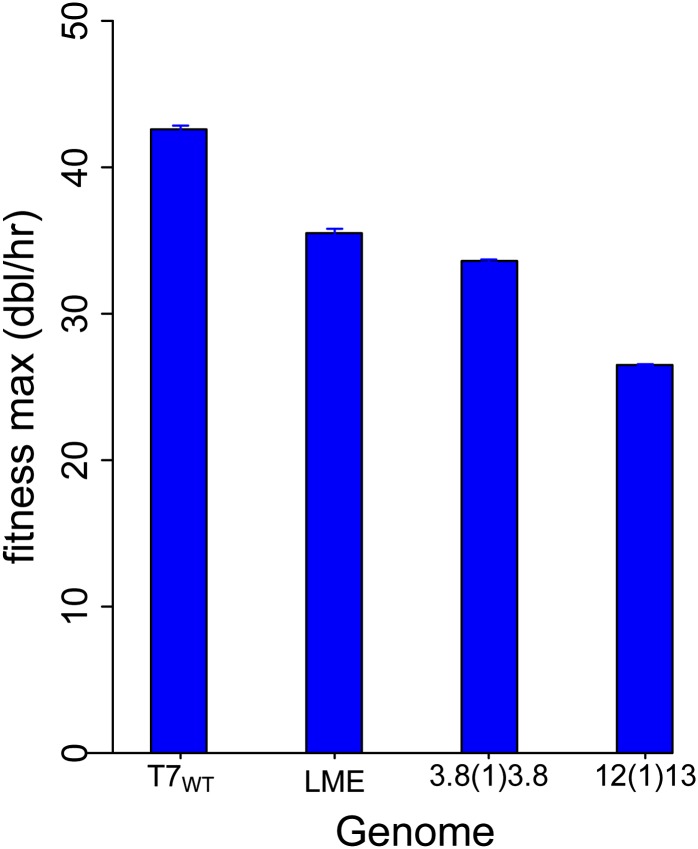
Evolutionary stable attenuation levels of T7 genomes with different types of gene rearrangements. All maximum fitness values (vertical axis) are substantially below that of the evolved wild-type (42.6) shown as the left-most bar. Error bars represent 1 std err and are barely visible in all cases. Note that fitness maxima are expressed as log(2), so major fitness differences are only slightly different visually.

The observed fitness evolution of T7:LME offers mixed support for the prediction that fitness will continue increasing indefinitely toward the wild-type value. Although molecular and fitness evolution continued beyond 200 generations, the rate of fitness increase was small, and extrapolation of the current trajectory would suggest a fitness limit well below that of wild-type. Even at its current fitness, this genome is the least attenuated of the 3 genomes studied here (Figure 7).

## Discussion

This study addressed several aspects of viral attenuation by genome rearrangement, a method of attenuation that was first developed for the eukaryotic virus VSV (Ball *et al.* 1999; Flanagan *et al.* 2001). Genome rearrangement has been used in a VSV-based vaccine against HIV-1 (Clarke *et al.* 2007; Cooper *et al.* 2008) and recently in an influenza vaccine (Pena *et al.* 2013), both moderately small-genome RNA viruses. The fact that genome rearrangement also attenuates the larger, double-stranded DNA virus T7 suggests that it may be a broadly applicable mechanism in viruses. Not all rearrangements of VSV exhibited strongly reduced fitness (Novella *et al.* 2004), and an exciting avenue for further exploration is to understand and predict which types of rearrangements are most debilitating.

Our specific focus is the evolutionary stability of attenuation—whether the attenuated virus will readily evolve to greater fitness across tens to hundreds of generations of adaptation. We equated the degree of attenuation with a quantitative reduction in viral fitness, a measure suitable for many purposes. Only one previous study has evaluated the evolutionary stability of attenuation by genome rearrangement (Springman *et al.* 2005), and the present study advances that understanding on several fronts. The virus used here was the bacteriophage T7. Following attachment to the host, the rate-limiting step in the T7 life cycle is expression of the phage RNAP gene. In two of the three adaptations performed, the RNAP gene was displaced to delay entry and thus delay expression of the RNAP gene. In the other design, the RNAP gene was in its wild-type location but early and late genes exchanged positions by ~20 kb.

The rearranged genome with RNAP gene in its wild-type location but with an exchange of other early-region genes with some late-region genes was considered to be the most likely to recover high fitness because there was no apparent delay in genome entry and expression of the phage RNAP gene. This *a priori* expectation does not neglect the imbalance in gene expression that ensues from the atypical gene order, but it does assume that those imbalances can be corrected by compensatory evolution. Despite this expectation, the genome realized only a small fitness increase during 1800 generations, and the final fitness was substantially lower than that of wild type. The implication is that many modifications of genome order can achieve attenuation for long periods of evolution, even gene orders that retain the natural order of the elements controlling entry and timing of gene expression. Furthermore, the fact that gene order has major effects on growth rate even when genome entry is normal suggests that the consequence of altered gene order probably extends to fitness measures that are not tied to a short generation time.

The ability to predict the evolutionary stability of attenuation and to predict the final fitness attained by an attenuated genome relies on understanding how fitness can be restored. The main basis for prediction in two experiments here was displacement of the RNAP gene downstream of its wild-type location. The phage RNAP is central to the phage life cycle. The main effects attributed to RNAP gene displacement are (1) a delay in its expression due to delayed entry, and (2) abnormal levels of expression due to autoamplification (the RNAP gene expresses itself when it is placed downstream of phage promoters). The former effect is easy to quantify with existing data, the latter more difficult. Furthermore, it is to be expected that displacement of the RNAP gene will have other effects; the question is how successful are predictions based on this limited perspective.

In experiments with a genome whose RNAP gene was displaced 23 kb, one prediction met with partial success−the evolution of an early entry mutation—but the predicted benefit of a downstream promoter was not observed. Yet even the predicted benefit of an early entry mutation was itself merely an observation from other studies extended to this study, not a theoretically or computationally derived result.

Another experiment relied on predictions from the virtual life cycle model of T7 that short displacements of the RNAP gene should have little fitness effect. The observed life cycle delay of such a construct was considerable, implying a serious fitness defect. Consequently, this genome was considered to be a good candidate for recovery of greater fitness because of the contrasting observations and virtual model prediction. In fact, final fitness remained depressed 9 dbl/hr below that of the evolved wild-type; with a 15-min generation time for both phages, the difference corresponds to a nearly fivefold difference in number of offspring. Our results suggest that prediction of the evolutionary response to genome rearrangement is challenging, even in well-studied systems, but not without some success.

The evolutionary stability of rearranged T7 genomes may be contrasted with an alternative means of attenuation, the use of rare codons that do not alter protein sequence to maintain lower levels of protein production (Burns *et al.* 2006, 2009; Mueller *et al.* 2006; Coleman *et al.* 2008). Work on poliovirus and T7 have both shown that different forms of engineered suboptimal codon usage can attenuate quantitatively to arbitrary levels. The question is then whether these attenuations are “permanent.” Although only a single codon-attenuated T7 genome has been tested (Bull *et al.* 2012), adaptation for ~1000 generations recovered considerable fitness with only a few mutations, the implication being that nearly full fitness is recoverable, albeit slower than the rate of fitness improvement observed in other T7 adaptations. Recovery of attenuated poliovirus was also observed within bounds (Burns *et al.* 2006), but the poliovirus work is difficult to evaluate because the virus was not pre-adapted to the growth conditions. If the T7 results generalize, genome rearrangement may be far superior to codon de-optimization as a lasting means of attenuation. One drawback of rearranged genomes, however, is that they carry all the genetic information to restore the wild-type virus through recombination. It must therefore be assured that the probability of such recombination is miniscule.

## Supplementary Material

Supporting Information
