# Supplementary material for: Evolutionarily Stable Attenuation by Genome Rearrangement in a Virus
Source: G3 (Bethesda). 2013 Aug 1;3(8):1389–97. doi: 10.1534/g3.113.006403 (PMC3737178; doi:10.1534/g3.113.006403)
Supplement: Supporting Information [file supp_3_8_1389__index.html]

Evolutionarily Stable Attenuation by Genome Rearrangement in a Virus — Supporting Information 

# Evolutionarily Stable Attenuation by Genome Rearrangement in a Virus

## Supporting Information for Cecchini *et al.*, 2013

**Files in this Data Supplement:**

- File S1 - Template genome files (.fasta, 152 KB)
